# Engineering a BMP2–risedronate complex with sustained release for osteoporosis therapy

**DOI:** 10.1007/s12272-025-01568-8

**Published:** 2025-09-24

**Authors:** Sang-Ok Jeon, Shrawani Lamichhane, Dong-Ho Oh, Jo-Eun Seo, Vinit Raj, Sangkil Lee

**Affiliations:** 1 Department of Pharmaceutical Engineering, Inje University, 197 Inje-Ro, Gimhae-Si, Gyeongsangnam-Do Republic of Korea; 2 College of Pharmacy, Chung-Ang University, 84 Heukseok-Ro, Dongjak-Gu, Seoul, Republic of Korea

**Keywords:** Bone morphogenetic protein 2 (BMP2), Bisphosphonates and ion-paring, BMP2–risedronate complex, Sustained release, Osteoblastic differentiation, Ectopic bone formation

## Abstract

Human bone morphogenetic protein 2 (BMP2) is a key inducer of osteogenesis; however, conventional delivery systems often result in burst release and adverse effects. To address this limitation, we fabricated an ionic complex of BMP2 with the clinically used osteogenesis inducer risedronate drug through an ion-pairing method. Fourier-transform infrared spectroscopy and ultra-resolution imaging confirmed the electrostatic interactions between BMP2 and risedronate. The resulting BMP2—risedronate complex exhibited a uniform particle size of 326 ± 149 nm, a polydispersity index of 0.2–0.3, and a zeta potential of − 38.08 ± 0.12 mV. The complex demonstrated a high complexation efficiency (˃ 95%). In vitro bioactivity studies using C2C12 myoblast cells demonstrated that BMP2, combined with risedronate, effectively induced osteoblastic differentiation. Furthermore, incorporation of the complex into 1.5% hyaluronic acid (HA) and 1% HA–19% poloxamer 407 (P407) hybrid hydrogel mitigated rapid release and retention, achieving a controlled release profile. In vivo studies confirmed that fabricated BMP2—risedronate complex loaded in hydrogels promotes sustained BMP2 release and, in combination with risedronate, enhances osteogenic efficacy. These findings suggest that the BMP2–risedronate ionic complex is a promising candidate for localized BMP2 delivery in osteogenesis therapy.

**Supplementary Information:**

The online version contains supplementary material available at 10.1007/s12272-025-01568-8.

## Introduction

Bone is a dynamic tissue that undergoes continuous remodeling through the coordinated actions of osteoclasts and osteoblasts in response to mechanical and biochemical cues. Bone morphogenetic proteins (BMPs), a subfamily of the transforming growth factor-β (TGF-β) superfamily, play a central role in osteoinduction by stimulating osteoblast differentiation, mineralization, and survival. Recent evidence also indicates that BMPs regulate osteoclastogenesis and coupling between resorption and formation, thereby maintaining bone homeostasis (Bordukalo-Nikšić et al. 2022a; Wu et al. 2024). Approximately 30 types of BMPs have been identified and characterized to date (Lowery and Rosen 2018). Among these, the U.S. Food and Drug Administration has approved two BMPs with osteoinductive properties, and BMP2 is widely used for the treatment of nonunion tibial and lumbar vertebral fractures, as well as sinus lift augmentation (Buell and Shaffrey 2021).

BMP2 is a homodimeric glycosylated protein consisting of 114 amino acids (aa) with a molecular weight of approximately 32 kDa (On et al. 2023). Human BMP2 is synthesized as a 396-aa preproprotein composed of a 23-aa signal peptide, a 259-aa prosegment, and a 114-aa mature region. Proteolytic cleavage removes the propeptide, yielding a mature homodimeric structure with seven conserved cysteine residues that form a characteristic knot-like structure (Fig. S1A). The BMP content in native cortical bone is low, typically ranging from 1 to 5 μg per gram (i.e., 1 to 5 μg/kg), depending on the particle size and extraction method (Pietrzak and Ali 2015). BMP2 is a basic protein with an isoelectric point of 8.2 ± 0.4 and exhibits high hydrophobicity (Pennec et al. 2024). It displays unusual solubility properties and is poorly soluble under physiological conditions because its residues are nearly electronically neutral at pH 7.2. Luca et al. (2010) investigated the effect of formulation PH on the physical stability of recombinant BMP2 and reported that the osteogenic protein tends to aggregate at pH 6.5 due to a decreased solubility compared with lower pH conditions.

Despite its strong osteogenic capacity, the clinical application of exogenous BMPs is limited by constraints such as a short half-life and rapid clearance from the site of application (Yan et al. 2018). To compensate for these limitations, high doses of BMPs are often required. However, such doses are associated with adverse effects, including immune responses, reactive oxygen species generation, excessive bone formation, reduced BMP2 activity, and increased treatment costs (Annamalai et al. 2018; Wang et al. 2021; Xu et al. 2021b). Consequently, numerous strategies have been developed to design effective delivery systems that provide controlled, site-specific release of BMP2. Controlled-release systems enhance BMP2 bioactivity and promote bone ossification. Collectively, these systems reduce the need for high doses and, consequently, the risk of complications. For example, Jeon et al. (2008) and La et al. (2010) demonstrated that BMP2-loaded poly(lactic-co-glycolic acid) (PLGA) nanosphere in fibrin gel provided improved bioactivity through sustained release (Luginbuehl et al. 2004). Their findings indicate that long-term delivery of BMP2 promotes more effective bone formation than short-term delivery at equivalent doses. Therefore, rational selection and design of BMP2 delivery systems are critical to achieving optimal biological effects and initiating new bone repair mechanisms. The release profile and bioactivity of BMP2 depend on interactions between the loaded protein and the delivery matrix, as well as the physicochemical properties and the dynamic properties of the carrier under physiological conditions. Novel approaches such as BMP2 immobilization, which involve covalent or noncovalent binding to a carrier matrix composed of biocompatible materials, including natural, synthetic, and inorganic polymers, as well as their composites, have provided new platforms for predictable, site-specific delivery.

Considerable efforts have been directed toward developing a novel delivery system for osteogenic proteins using various materials (Howard et al. 2022; Wu et al. 2022; Zhen et al. 2022; Sun et al. 2025). Numerous researchers have fabricated delivery systems using novel biodegradable polymers capable of effectively incorporating BMP2 through various approaches, including microencapsulation, polymeric film coating on implant surfaces (Howard et al. 2022), absorption into sponge/scaffold (Marshall et al. 2024a, 2024b; Kim et al. 2025), surface modification of microspheres or beads (Wu et al. 2022), and interaction with biodegradable polyelectrolytes (Shah et al. 2011; Wang et al. 2015). However, stress-induced structural changes and loss of biological activity remain challenges in the design of the BMP2 delivery systems. Several studies have been conducted to develop particulate systems containing BMP2, based on its decreased solubility under physiological conditions and its binding affinity for heparin (Hettiaratchi et al. 2020; Kim et al. 2020). These particulate systems act as carriers that incorporate therapeutic BMP2 and regulate its release profile. Ionic complex-based delivery systems are formed through electrostatic interactions between oppositely charged surfaces, which are controlled by the pH of the reactant solution. At specific pH levels, the particles acquire opposite surface charges, facilitating ion pairing via electrostatic interactions.

In this study, we hypothesized that antiresorptive bisphosphonates (BPs) could serve as an ion-pairing counterpart to control and prolong the release of BMP2 via electrostatic interaction, thereby enhancing its therapeutic potential for osteoporosis. BP derivatives, such as inorganic pyrophosphate, inhibit mineral deposition and are widely used in the treatment of osteoporosis, Paget's disease, hypercalcemia of malignancy, and bone metastasis (Ralston 2020; Eshaghi et al. 2025). Nitrogen-containing BPs effectively suppress osteoclast activity and induce apoptosis by inhibiting farnesyl diphosphate synthase in the mevalonate pathway (Xu et al. 2024). Among these, risedronate is a widely used first-line treatment for osteoporosis (Wang et al. 2018).

To test this hypothesis, we fabricated a novel BMP2–risedronate complex using an ion-pairing technique. The BMP2–risedronate ionic complex was characterized in terms of particle size, polydispersity index (PI), morphology, and complexation efficiency. Furthermore, Fourier-transform infrared spectroscopy (FT–IR), circular dichroism (CD), and sodium dodecyl sulfate–polyacrylamide gel electrophoresis (SDS–PAGE) were employed to assess the conformational integrity of BMP2 within the BMP2—risedronate ionic complex. In vitro release studies were conducted in various release media, and the bioactivity of the BMP2—risedronate ionic complex was evaluated using murine undifferentiated C2C12 myoblast cells. Osteoblastic differentiation was assessed using real-time polymerase chain reaction (PCR) to determine the relevant expression of mRNA. Finally, the in vivo osteogenic efficacy of the BMP2—risedronate ionic complex was investigated using an ectopic bone formation model in Sprague–Dawley (SD) rats and further analyzed by micro-computed tomography (μCT). To our knowledge, this is the first report on the fabrication and investigation of the osteogenic efficiency of a BMP2–risedronate ionic complex.

## Materials and methods

### Materials

Recombinant human BMP2 was kindly provided by Daewoong Pharm. Co., Ltd. (Yongin, Gyeonggi-do, South Korea). Risedronate sodium monohydrate (Mw 323.1) was obtained from Dongwoo Syntech Co., Ltd. Hyaluronic acid (HA) was purchased from DHP Korea (Seoul, Korea), and poloxamer 407 (P407) was purchased from BASF (Wyandotte Corp., Parsippany, NJ). All other chemicals were of analytical grade.

### Analysis of BMP2 and risedronate

#### Analysis of BMP2

The concentration of BMP2 was quantified using a human BMP2 ELISA kit (RHF913CKX, Antigenix America Inc., New York, NY, USA) according to the manufacturer's instructions. Dissociated BMP2 from the BMP2–risedronate ionic complex was measured using an HPLC system (YL9100, Young Lin Instrument Co., Ltd., Anyang, Korea). Separation was performed on a C_4_ column (Vydac 218TP, 4.6 × 250 mm, 5 μm) with a flow rate of 0.2 mL/min and an injection volume of 20 μL. The effluent was monitored at 214 nm. The mobile phase consisted of 0.1% trifluoroacetic acid (TFA) in distilled water and 0.1% TFA in acetonitrile (JT Baker, Phillipsburg, NH) (70:30, v/v). A gradient elution was applied, with the mobile phase adjusted from 20:80 (v/v) between 8 and 20 min, then returned to 70:30 (v/v) after 20 min.

#### Analysis of risedronate

The concentration of unreacted risedronate during the ion-pairing process was determined by HPLC using ion-pair chromatography with a C_18_ reverse-phase column (ZORBAX Eclipse XDB-C_18_, 4.6 × 250 mm, 5 μm). The flow rate was 1.0 mL/min, the injection volume was 20 μL, and the effluent was monitored at 262 nm. The mobile phase comprised acetonitrile and an aqueous solution containing 5 mM tetrabutylammonium bromide and 5 mM sodium pyrophosphate (pH 7.0) in a 22:78 (v/v) ratio.

### Fabrication of BMP2–risedronate ionic complex

The BMP2–risedronate ionic complex was prepared as outlined in Fig. S2. Separate aqueous solutions of BMP2 (1 mg/mL) and risedronate (1.25 mg/mL) were prepared, and the pH was adjusted to 5.7–5.8 with 1 N sodium hydroxide (NaOH). The BMP2 solution was slowly injected into the risedronate solution using a peristaltic syringe pump (KDS 100, Kd Scientific, USA) with a 23-gauge needle at a rate of 0.17 mL/min, while the mixture was stirred with a magnetic bar under mild conditions for 3 h at room temperature (RT). The resulting cloudy solution was centrifuged at 12,000 rpm for 10 min (Smart R17, Hanil Science, Seoul, Korea), and the supernatant was collected for quantification of unreacted BMP2 and risedronate. The BMP2–risedronate ionic complex was lyophilized and stored at − 20 ℃ until further use.

### Characterization of BMP2–risedronate ionic complex

#### Particle size and PI of BMP2—risedronate ionic complex

Particle size and PI of the BMP2—risedronate ionic complex were measured using dynamic light scattering (ZetaPALS, Brookhaven Instrument, Holtsville, NY, USA). The complex suspension was diluted 20-fold with 0.1% Tween^®^ 80 solution, and measurements were performed in triplicate.

#### Structural integrity and secondary structure analyses of BMP2 in an ionic complex

FT–IR study was performed to characterize complex formation and secondary structural changes in BMP2 during the complex preparation. Samples were mixed with KBr powder and pressed into disks under 7.54 ton/cm^2^ of pressure. Spectra were recorded over the range of 400–4000 cm^−1^ using an FT–IR spectrophotometer (Varian 1000 FT-IR, Varian, USA), with 32 scans averaged at a resolution of 4 cm^−1^. Spectra were analyzed in the amide I region (1600–1690 cm^−1^) using second-derivative methods in Omnic software (Nicolet, Waltham, MA). Gaussian band profiles were applied to fit the inverted second-derivative FT–IR spectra. The area of each component in the amide I region was calculated as the ratio of the component peak area to the sum of all peak areas in the amide I band, and the results were expressed as percentages.

The structural integrity of dissociated BMP2 from the BMP2—risedronate ionic complex was assessed using CD spectroscopy in the far-ultraviolet (UV) region (190–250 nm) with a spectropolarimeter (Jasco J-715, JASCO, Tokyo, Japan). BMP2 at 6.54 μM (0.17 mg/mL) was placed in a 1-mm pathlength cuvette under a nitrogen atmosphere. Six scans were accumulated at a scan speed of 100 nm/min, and spectra were corrected against a background buffer. Secondary structure content was determined using the Jasco secondary structure estimation program.

To evaluate the effect of the preparation process on the BMP2 structural integrity, electrophoresis was performed using an XCell SureLock^®^ Mini-Cell gel system with NuPAGE^®^ Novex Bis–Tris pre-cast gel (4–12%, 1 mm) and NuPAGE^®^ MES SDS running buffer (Invitrogen GmbH, Karlsruhe, Germany). Non-reduced samples were prepared using Bolt™ LDS sample buffer, and reduced samples were prepared using Bolt™ sample reducing agent, following the manufacturer’s instructions. Samples containing 2 μg of BMP2 in a total volume of 20 μL were heated at 70 °C for 10 min, then centrifuged and loaded into the wells. Gels were run at 200 V for 35 min, and protein bands were visualized using Coomassie Brilliant Blue G-250 (Thermo Fisher Inc., MA, USA) and a silver staining kit (Komabiotech, Seoul, Korea).

#### Morphological analysis of BMP2—risedronate ionic complex

The morphology of the BMP2—risedronate ionic complex was characterized using transmission electron microscopy (TEM, H-7500, Hitachi Ltd., Japan) and scanning electron microscopy (SEM, S-4200, Hitachi Ltd., Japan).

For SEM image analysis, the complex suspension was dried on a substrate and coated with gold–palladium for 20 min to minimize surface charging. Samples were then examined under a field emission SEM to visualize surface morphology. For TEM image analysis, a drop of the complex suspension was placed on a carbon-coated copper grid. The excess solution was removed with filter paper, and the sample was dried for 10 min. TEM imaging was performed at magnifications of 50,000–200,000 × with an accelerating voltage of 80 kV.

High-resolution images were obtained using an optical microscope equipped with the CytoViva™ ultra-resolution imaging system (Aetos Technologies Inc., Auburn, AL, USA). Before imaging the BMP2—risedronate ionic complex, aliquots of BMP2 and risedronate solutions were individually placed on a glass slide as references, and their spectral profiles were recorded over a scanning range of 400–1000 nm, with the specific absorption wavelengths of each agent indicated by distinct colors. For imaging the BMP2–risedronate ionic complex, an aliquot of the complex suspension, diluted with distilled water, was placed on a glass slide, and its spectral profile was obtained over the same wavelength range. Finally, the image of the BMP2–risedronate ionic complex was visualized relative to the reference using a mapping process.

#### Calculation of complexation efficiency

To determine complexation efficiency, the reaction solution was centrifuged, and the concentration of BMP2 in the supernatant was measured using a human BMP2 ELISA kit. The complexation efficiency (percentage) of BMP2 was calculated using Eq. (1).

1 $${\text{Complexation}}\,{\text{ efficiency }}\left( \% \right) = \frac{{\left( {A - B} \right)}}{B} \times { 1}00$$

where A is the initial amount of BMP2 added to the formulation, and B is the amount of BMP2 remaining in the supernatant.

### Assessment of BMP2–risedronate complex formation using an in silico approach

To investigate potential interactions between BMP2 and risedronate, molecular docking was performed using AUTODOCK and VINA tools. Molecular docking generally enables the prediction of conformational binding between a ligand and its receptor, as well as the identification of interacting amino acid residues. The crystal structure of BMP2 (PDB ID: 3BMP) with a resolution of 2.70 Å was retrieved from the RCSB Protein Data Bank (https://www.rcsb.org). The protein structure was further refined using the Protein Preparation Wizard in the Schrodinger suite. Hydrogen atoms were added as required, and an extra co-crystallized water molecule was removed from the BMP2 structure. The risedronate ligand structure was retrieved from the PubChem database. The ligand was energy-minimized using density functional theory to obtain a stable molecular conformation. The ligand was then subjected to molecular docking with the BMP2 protein using the VINA tool. To ensure reliability, reproducibility, and validation, re-docking was performed using AUTODOCK. In the docking study, the ligand was treated as a rigid entity, whereas the protein was modeled as a flexible entity. Finally, the ligand–protein complex interactions were visualized using the BIOVIA Discovery Studio.

### Investigation of conformational stability of BMP2–risedronate complex by molecular dynamics (MD) simulation

MD simulation was employed to analyze the confirmation stability of the BMP2–risedronate complex in an explicit water solution over a defined period. The top-scoring docked complex of risedronate–BMP2 was submitted for MD simulation using YASARA Dynamics software on an LG Intel Core i5 CPU (64-bit enterprise version). The previously reported protocol was adopted with minor modifications for the MD simulation of the BMP2–risedronate ionic complex (Raj et al. 2021, 2022). Periodic boundary conditions were defined according to the size of the complex, and the simulation box was filled with water at a density of 0.997 g/mL. Sodium and chloride ions were randomly placed to achieve overall charge neutrality of the system. The temperature was initially set to 298 K and gradually increased to reach equilibrium. An AMBER03 molecular mechanics force field was assigned under reproduced physiological conditions at pH 7.4 and 0.9% NaCl. The system was subsequently submitted to an MD run at 1 ps pressure and 310 K for 73.1 ns. MD trajectories were recorded every 250 ps over 73.1 ns. All trajectories were analyzed, and root mean square deviations (RMSDs) and binding energies were plotted using Origin 2018.

### In vitro BMP2 release study

The in vitro release profile of BMP2 from the BMP2–risedronate ionic complex was evaluated in phosphate-buffered saline (PBS, pH 4.8 and 7.4) and simulated body fluid (SBF, pH 7.4) containing 0.05% bovine serum albumin and 0.02% sodium azide. The BMP2–risedronate ionic complex, containing approximately 125 μg of BMP2, was incubated in 7 mL of each condition at approximately 36.5 ℃. The samples were centrifuged at 12,000 rpm for 10 min at predetermined time intervals, and 5 mL of supernatant was collected, which was subsequently replaced with an equal volume of fresh buffer. Similarly, to evaluate the effect of HA hydrogel and hybrid hydrogel formulations on BMP2 release, 0.5%, 1%, 1.5%, and 2% of HA, along with various hybrid hydrogels (0.5% HA + 19.5% poloxamer, 1% HA + 19% poloxamer, 1.5% HA + 18.5% poloxamer, and 2% HA + 18% poloxamer), were included. The amount of BMP2 in the supernatant aliquots was measured using a human BMP2 ELISA kit.

### Rheological properties of HA and HA–P407 hybrid hydrogels

The rheological properties of HA hydrogels and HA−P407 hybrid hydrogels were evaluated using a rotational rheometer (MCR 302e, Anton Paar, Graz, AUS). Hydrogel samples were thoroughly mixed to obtain homogeneous formulations. The viscosity of each formulation was measured as a function of shear rate to assess shear-thinning behavior. Samples were subjected to a continuous ramp of shear rates ranging from 0.1 to 100 s^−1^ at 4 °C, RT, and 37 °C. Viscoelastic properties were evaluated by frequency sweep tests. Storage modulus (*G*′) and loss modulus (*G*′′) were measured over a frequency range at 10% strain within the linear viscoelastic region to ensure non-destructive testing. All measurements were conducted at 37 °C to assess the elastic and viscous behavior of the hydrogels under dynamic oscillatory conditions.

### Injectability study of HA and hybrid hydrogels

The injectability of various concentrations of HA and HA−P407 hybrid hydrogel was evaluated to determine its suitability for clinical administration, following the method described by Alves. Approximately 1 mL of each hydrogel formulation was loaded into a 1 mL syringe fitted with a 23-gauge needle. A constant force of 50 N was applied for 5 s to extrude the hydrogel. The injectability of each formulation was calculated as the percentage of the extruded volume relative to the original volume loaded into the syringe. Percentage injectability was determined using Eq. (2).

2 $${\text{Injectability }}\left( \% \right) = \frac{{Extruded \,amount \left( {{\text{mL}}} \right)}}{{Initial\, amount \left( {{\text{mL}}} \right)}} \times {1}00$$

Each formulation was tested in triplicate at 4 °C and RT to evaluate the effect of temperature on injectability.

### In vitro cell viability and potency of BMP2—risedronate ionic complex

#### C2C12 cell culture and viability assay

Murine C2C12 skeletal myoblasts were cultured in Dulbecco's Modified Eagle's Medium (DMEM) supplemented with 10% (v/v) fetal bovine serum (FBS), 100 U/mL penicillin, and 100 μg/mL streptomycin. Cells were maintained at 37 °C in a humidified 5% CO_2_ atmosphere, and the medium was replenished three times per week. Differentiation was induced in DMEM containing 2% FBS as the negative control or in the presence of samples. Culture media were changed daily until the alkaline phosphatase (ALP) activity was determined.

C2C12 cells were seeded in 96-well plates at 2 × 10^3^ cells/well. After 24 h, cells were incubated with samples diluted in DMEM containing 2% FBS for 2 days. Formazan crystals were dissolved in dimethyl sulfoxide and mixed for 5 min after incubation with thiazolyl blue tetrazolium bromide solution for 2 h. Absorbance was measured at 560 nm using a VERSAmax^®^ microplate reader (Molecular Devices, Sunnyvale, CA). Cell viability was expressed as a percentage of the control and measured in triplicate.

#### In vitro estimation of ALP activity of BMP2—risedronate ionic complex

C2C12 cells were seeded in 12-well plates at 1 × 10^4^ cells/well. After 24 h, the cells were incubated with the samples diluted in DMEM containing 2% FBS for 7 days. Cells were washed with PBS and lysed using RIPA buffer (Thermo Scientific, Waltham, MA, USA) supplemented with protease inhibitor. Lysates were centrifuged at 12,000 rpm for 10 min at 4 °C. Total protein content in the supernatant was quantified using a bicinchoninic acid (BCA) assay. ALP activity was measured using the LabAssay™ ALP kit (Wako Pure Chemical Industries, Ltd., Osaka, Japan) according to the manufacturer's instructions. Experiments were performed in triplicate. ALP activity was normalized to protein content and expressed as millimole per liter (mmol/L) of p-nitrophenol released per protein content.

#### In vitro osteogenic gene expression of BMP2—risedronate ionic complex

C2C12 cells were seeded in 12-well plates at 1 × 10^4^ cells/well. After 24 h, cells were incubated with samples diluted in DMEM containing 2% FBS for 3, 5, and 7 days. Total RNA was isolated using AccuZol™ reagent (Bioneer, Daejeon, South Korea) according to the manufacturer's instructions. RNA purity and concentration were measured using a NanoDrop (Thermo Fisher Scientific, Wilmington, DE). First-strand cDNA was synthesized from 1 μg of total RNA using oligo-dT18 primer and RevertAid™ M-MuLV reverse transcriptase (RevertAid™ First-strand cDNA Synthesis Kit, Fermentas, Hanover, USA). SYBR Green-based quantitative PCR was performed using the LightCycler^®^ 480 Real-Time PCR System (Roche Diagnostics, Meylan, France) and LightCycler^®^ 480 SYBR Green I Master Mix (Roche). Relative gene expression was calculated using the GAPDH-normalized 2^−△△Ct^. Primer sequences are provided in Table S1.

#### Alizarin red S staining

C2C12 cells were seeded in 96-well plates at 1 × 10^4^ cells/well. After 24 h, cells were incubated with samples in DMEM containing 2% FBS, ascorbic acid (50 µg/mL, Merck Millipore, St. Louis, USA), and β-glycerophosphate (10 mM, Merck Millipore, St. Louis, USA) for 21 days, with media replenished weekly. Cells were washed twice with PBS and fixed in neutral buffered formalin (10%, Merck Millipore, St. Louis, USA) for 30 min. After washing twice with distilled water, cells were stained with alizarin red S staining solution (40 mM, pH 4.2, Merck Millipore, St. Louis, USA). After incubating for 30 min, the alizarin red S solution was removed, and the cells were washed twice with distilled water. The stained cells were examined under a transmitted light microscope (Olympus, Tokyo, Japan) and images captured using a digital camera.

#### Immunocytochemistry for type I collagen expression

After culture, the media was removed, and cells were gently washed once with PBS. Cells were fixed with 4% paraformaldehyde (Merck Millipore, St. Louis, USA) for 10 min at RT, followed by two PBS washes. For permeabilization, cells were incubated with 0.5% Triton X-100 in PBS for 5 min at RT. To reduce background fluorescence, the cells were blocked with 1% normal goat serum in PBS for 1 h at RT. The primary antibody against type I collagen (ABclonal, MA, USA) was diluted in 1% normal goat serum in PBS and applied to the cells, followed by overnight incubation at 4 °C. The next day, cells were washed three times with PBS for 5 min each. Secondary antibody staining was performed using goat anti-rabbit IgG (H + L) cross-adsorbed, Alexa Fluor™ 488 (Invitrogen, MA, USA) diluted in 1% normal goat serum in PBS and incubated for 1 h at RT in the dark. The cells were then washed three times with PBS for 5 min each. Nuclear staining was performed using Hoechst 33,342 (Invitrogen, MA, USA) for 20 min at RT, followed by three additional washes with PBS, and the samples were imaged using a confocal laser scanning microscope (Carl Zeiss, Oberkochen, Germany).

### In vivo ectopic bone formation

#### Surgical procedure

An injectable hydrogel was employed to deliver the formulations for ectopic bone formation. The hydrogels were classified into two types: HA and HA–P407 hybrid hydrogels. Owing to the thermoreversible character of P407, its hydrogel was prepared with homogeneous stirring at 4 ℃, and the hydrogels were mixed with the BMP2—risedronate ionic complex immediately before injection.

The 7-week-old male SD rats were weighed and anesthetized with 2.5% tribromoethanol (300 mg/kg). Thirty animals were divided into five experimental groups (n = 5): negative control, BMP2 in HA, BMP2—risedronate ionic complex in HA, BMP2 in HA−P407 hybrid hydrogel, and BMP2—risedronate ionic complex in HA−P407 hybrid hydrogel. The injection site was shaved, and 100 μL of hydrogel was administered into the left hind limb muscle for the HA groups and the right hind limb muscle for the HA−P407 hybrid hydrogel groups. All samples contained the equivalent of 105 μg of BMP2 per hydrogel, and hydrogels without BMP2 or risedronate served as negative controls. Rats were fed ad libitum following the injection. Four weeks after injection, ectopic bone formation at the injection site was confirmed using computed tomography (CT), after which the rats were sacrificed by CO_2_ asphyxiation. The hind limb muscles, including the injection site, were then excised and fixed in 10% formalin solution (Sigma, St. Louis, MO) for subsequent analysis. All animal care and surgical procedures were performed following the guidelines approved by the Busan National University Hospital Animal Ethics Committee (PNUH-2014-059).

#### In vivo CT scanning of ectopic bone formation

To confirm the presence of ectopic bone at the injection site 4 weeks after injection, in vivo CT images of the rat hind limbs were acquired using a small-animal imaging system (Inveon, Siemens Preclinical Solutions, Knoxville, TN, USA). Anesthesia was induced and maintained with isoflurane inhalation (IFRAN LIQ, Hana Pharm. Co., Korea), and the animals were positioned in the scanner. CT images were captured at 80 kV and 500 A, with an exposure time of 800 ms and a spatial resolution of 40 µm. Using image analysis software, the tomographic images were reconstructed into volumetric models (Inveon Research Workplace (IRW) version 2.2, Siemens Medical Solutions USA Inc., Knoxville, USA). For morphometric analysis, newly generated bone at the injection site was selected as the volume of interest using auto-interpolation, and mineral bone volume (mm^3^) was calculated using IRW software. The results were further converted to bone mineral density (BMD, mg/cm^3^).

#### Histological evaluation of the injection site

Four weeks after injection, the rats were sacrificed by CO_2_ asphyxiation, and the hind limb muscles, including the injection site, were excised. The specimens were fixed in a 10% neutral buffered formalin solution for 5 days and subsequently decalcified in ethylenediamine tetraacetic acid. The decalcified samples were embedded in paraffin and sectioned at a thickness of 4 µm. Hematoxylin and eosin (H&E) staining was performed, and the stained sections were observed using an inverted microscope (Olympus-X-71, Olympus America Inc., USA).

### Statistical analysis

All quantitative data were presented as means ± standard deviation. Statistical analysis was performed using one-way analysis of variance (ANOVA). The significance of differences among groups was determined using the Student–Newman–Keuls test and Tukey’s multiple comparison method. A *p-*value < 0.05 was considered statistically significant.

## Results

### Characterization of the BMP2—risedronate ionic complex

**Fig. 1:**
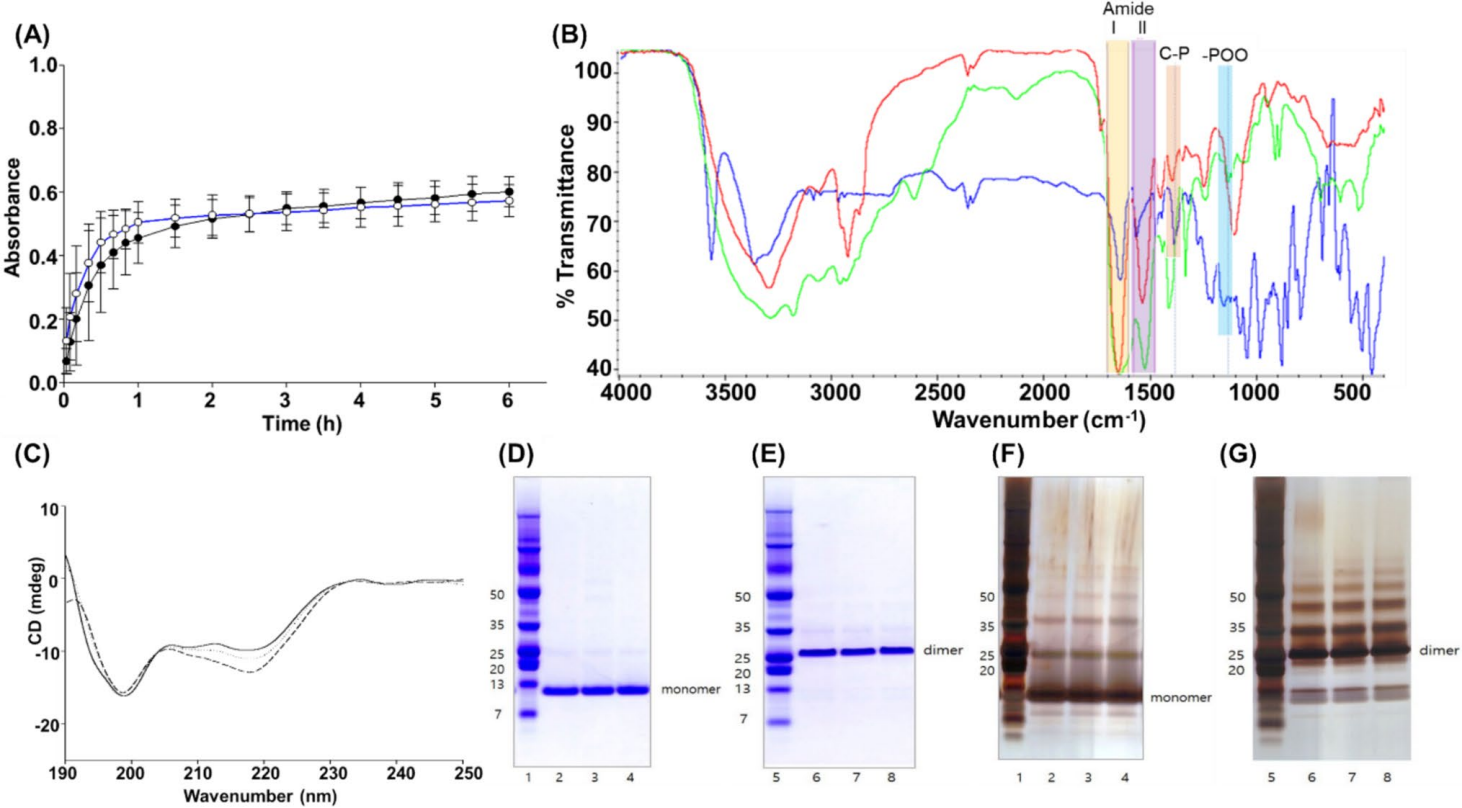
Characterization of the complex. **A** Effect of temperature on the reaction kinetics of the complex. ●, incubation at RT for 6 h; ○, incubation at RT for 1 h followed by 4 ℃ for the remaining 5 h. **B** Representative FT–IR spectra of the BMP2—risedronate complex (green), free BMP2 (red), and risedronate (blue). **C** CD spectra of dissociated BMP2 after ion-pairing process with (solid line) or without (dotted line) lyophilization, compared with native BMP2 in 1 mM HCl (short-dashed line). Polyacrylamide gels stained with Coomassie blue (**D**, **E**) and silver nitrate (**F**, **G**). Samples were analyzed under reducing (**D**, **F**) and nonreducing conditions (**E**, **G**). Lanes 1 and 5, marker standard; lanes 2 and 6, native BMP2; lanes 3 and 7, dissociated BMP2 from the complex without lyophilization; lanes 4 and 8, dissociated BMP2 from the complex with lyophilization

The mean diameter and PI of the BMP2—risedronate ionic complex in an aqueous solution were 326 ± 149 nm and 0.2–0.3, respectively, with a zeta potential of – 38.08 ± 0.12 mV. To determine the reaction termination time, the absorbance at 400 nm was monitored under different preparation conditions. The results indicated that complex preparation was completed after approximately 3 h, and temperature had no effect on the reaction termination time (Fig. 1A). The number of unreacted components was used to calculate the reaction ratio and complexation efficiency. The molar ratio of the components was determined to be 1:20, and the complexation efficiency of BMP2 was approximately > 95%.

FT–IR and CD analyses were conducted to assess potential conformational changes in BMP2 structure during the ion pairing process. Furthermore, SDS-PAGE was employed to evaluate the stability of BMP2 following dissociation of the BMP2–risedronate ionic complex under both reducing and nonreducing conditions. FT–IR spectra of BMP2, risedronate, and the BMP2—risedronate ionic complex were obtained to examine interactions between BMP2 and risedronate. As shown in Fig. 1B, the FT-IR spectrum of risedronate (red) showed characteristic peaks at approximately 1,134 cm⁻^1^ and 1,385 cm⁻^1^, corresponding to the symmetric stretching vibration of the ─POO^−^ (phosphate ester) group and the vibration of the C─P group, respectively (Fig. S1B). In contrast, the FTIR spectra of the BMP2—risedronate ionic complex showed the disappearance of the ─POO^−^ peak, suggesting interactions between the negatively charged phosphate ester groups of risedronate and the positively charged amine groups of BMP2. Furthermore, FT-IR provides insight into protein conformational changes and structural stability. Amide I and II bands are the most prominent vibrational bands used to characterize protein structure (Yang et al. 2022; Baghel et al. 2024). The amide I band (1600–1700 cm^−1^) arises from the sum of overlapping component bands (α-Helix, β-Sheet, Turn, and random), primarily attributed to C═O stretching vibration of the polypeptide backbone (80%), and its band shape is highly sensitive to protein secondary structure (Li et al. 2019; Yang et al. 2022). In contrast, the amide II band (1470–1570 cm^−1^) is associated with C─N stretching and N─H bending vibrations (Kong and Yu 2007; Ji et al. 2020). For the free BMP2 spectrum (red), a sharp peak at 1656 cm^−1^ was observed, corresponding to the amide I band (C═O stretching) of the α-helix structure, while a strong band at 1627 cm^−1^ was attributed to the β-sheet structure. Additionally, bands at 1672 and 1646 cm^−1^ were assigned to unordered structures and β-turn, respectively. In the BMP2–risedronate ionic complex, the presence of a broader amide I band along with a weaker amide II band suggests successful complexation with risedronate.

CD spectroscopy was employed to investigate conformational changes, structural stability, and interactions involving BMP2, either with other proteins or with risedronate. Absorption in the far-ultraviolet region (178–260 nm) corresponds to the peptide bond, with signals around 220 nm and 190 nm representing the n → π* and π → π* transitions, respectively. The change in CD spectra in this region is sensitive to protein conformation. Although CD does not provide absolute structural information, it is highly effective in detecting conformational changes in proteins. BMP2 dissociated from the BMP2—risedronate ionic complex was compared with native BMP2 to assess conformational alterations. A centrifugal filter device (Amicon Ultra, 3000 MWCO, Millipore Corp., USA) was used to remove excipients in the formulation that could interfere with CD measurements. After concentrating BMP2 and eliminating additives, the protein buffer was exchanged with 1 mM HCl (pH 4). CD spectra of all samples were recorded in the far-UV range (190–250 nm) under identical buffer conditions. The spectra are shown in Fig. 1C, exhibiting minima at 198 and 218 nm in all cases. These findings are consistent with experimental data (Lin et al. 2016; Liu et al. 2023; Yu et al. 2024). Moreover, the shape of the spectra showed a typical protein structure predominantly composed of β-sheet. The quantitative content of each secondary structure was estimated from CD spectra using the Jasco secondary structure estimation program, as presented in Table 1. Furthermore, the lyophilization process was effective in preserving BMP2 conformation, with minimal variation between samples (solid line vs. dotted line).

**Table 1:** Comparison of secondary structure content (%) of native BMP2 and BMP2 dissociated from the complex by circular dichroism

Secondary structure	Native BMP2 in 1 mM HCl (%)	BMP2 in complex before lyophilization (%)	BMP2 in complex after lyophilization (%)
α-Helix	2.6	0.0	0.0
β-Sheet	51.9	55.1	50.5
Turn	6.3	2.6	4.0
Random	39.2	42.3	45.5
**Total**	100.0	100.0	100.0

To evaluate the structural integrity and consequent BMP2 aggregate formation in the BMP2–risedronate ionic complex, dissociated BMP2 was compared with the native one using PAGE. PAGE is a traditional and reliable method for determining protein aggregation (Zhai et al. 2025). The results demonstrated that BMP2 maintained its homodimer structure, indicating that structural stability was maintained regardless of the lyophilization process. Following dissociation under nonreducing conditions, BMP2 released from the BMP2—risedronate ionic complex exhibited a molecular weight of approximately 26 kDa, comparable to that of the native form (Fig. 1E, G). Similarly, resolving the BMP2—risedronate ionic complex under reducing conditions yielded monomeric BMP2 with a molecular weight of approximately 13 kDa, consistent with the native form under the same conditions. Moreover, the structural stability of BMP2 in the complex was maintained independently of the lyophilization process (Fig. 1D, F).

**Fig. 2:**
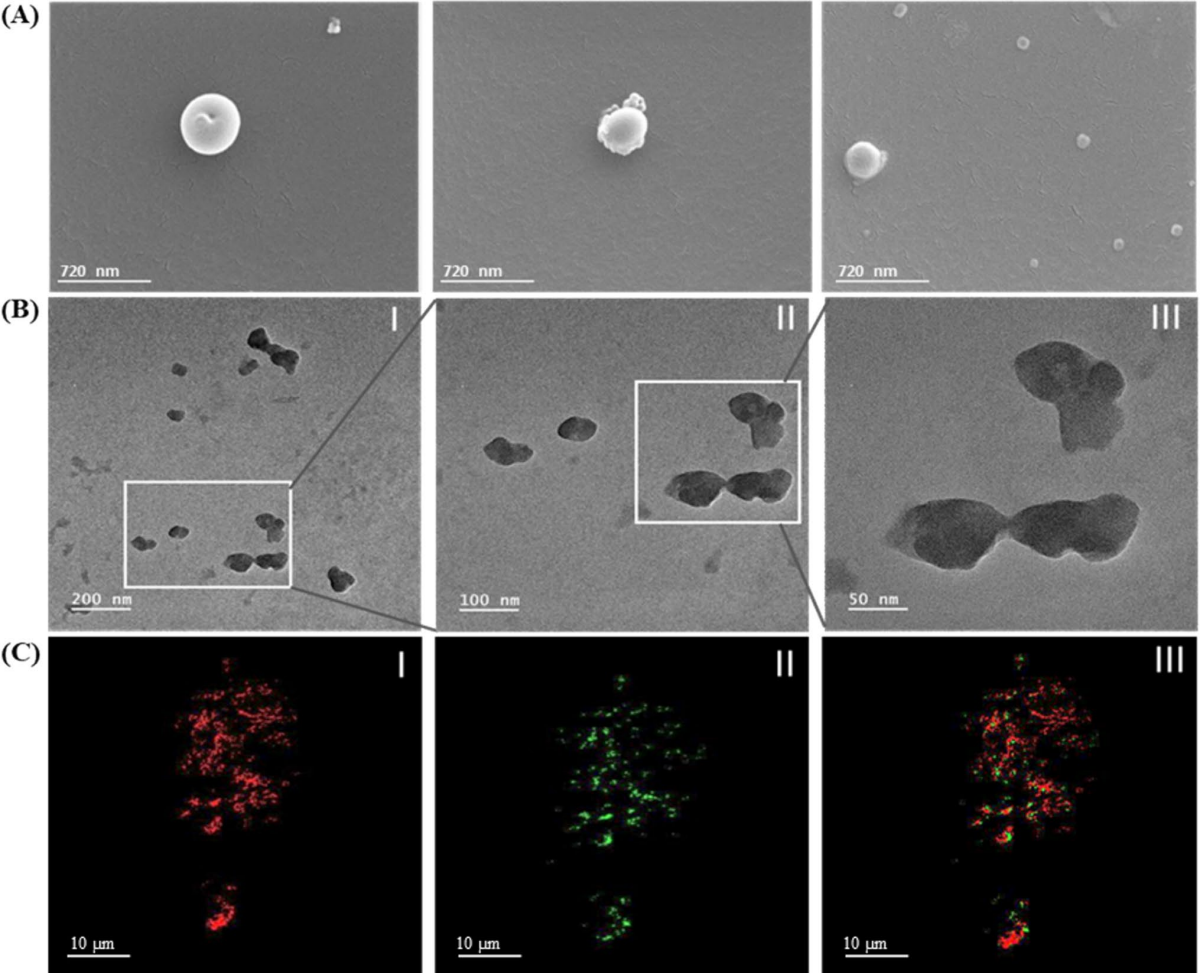
Characterization of surface architecture and morphology of the BMP2–risedronate complex. **A** SEM images at 25,000× magnification. **B** TEM images at 5000× magnification. **C** Hyperspectral microscopic images of BMP2–risedronate complex using Cytoviva^TM^instrument: (I) BMP2 (red), (II) risedronate (green), and (III) mapping distribution of BMP2 and risedronate in the complex

The morphology of the BMP2–risedronate ionic complex was further examined using electron microscopy. The complex had a spherical shape with a smooth surface and uniform size distribution, consistent with the particle size analysis (Fig. 2A, B). High-resolution optical microscopy with Cytoviva™ allowed the visualization of BMP2, risedronate, and the BMP2–risedronate ionic complex at the submicron level without the use of special staining techniques. In the complex, BMP2 and risedronate were represented as red and green colors, respectively, as shown in Fig. 2C.

### Molecular docking of risedronate with BMP2 protein to reveal the potential molecular interactions

**Fig. 3:**
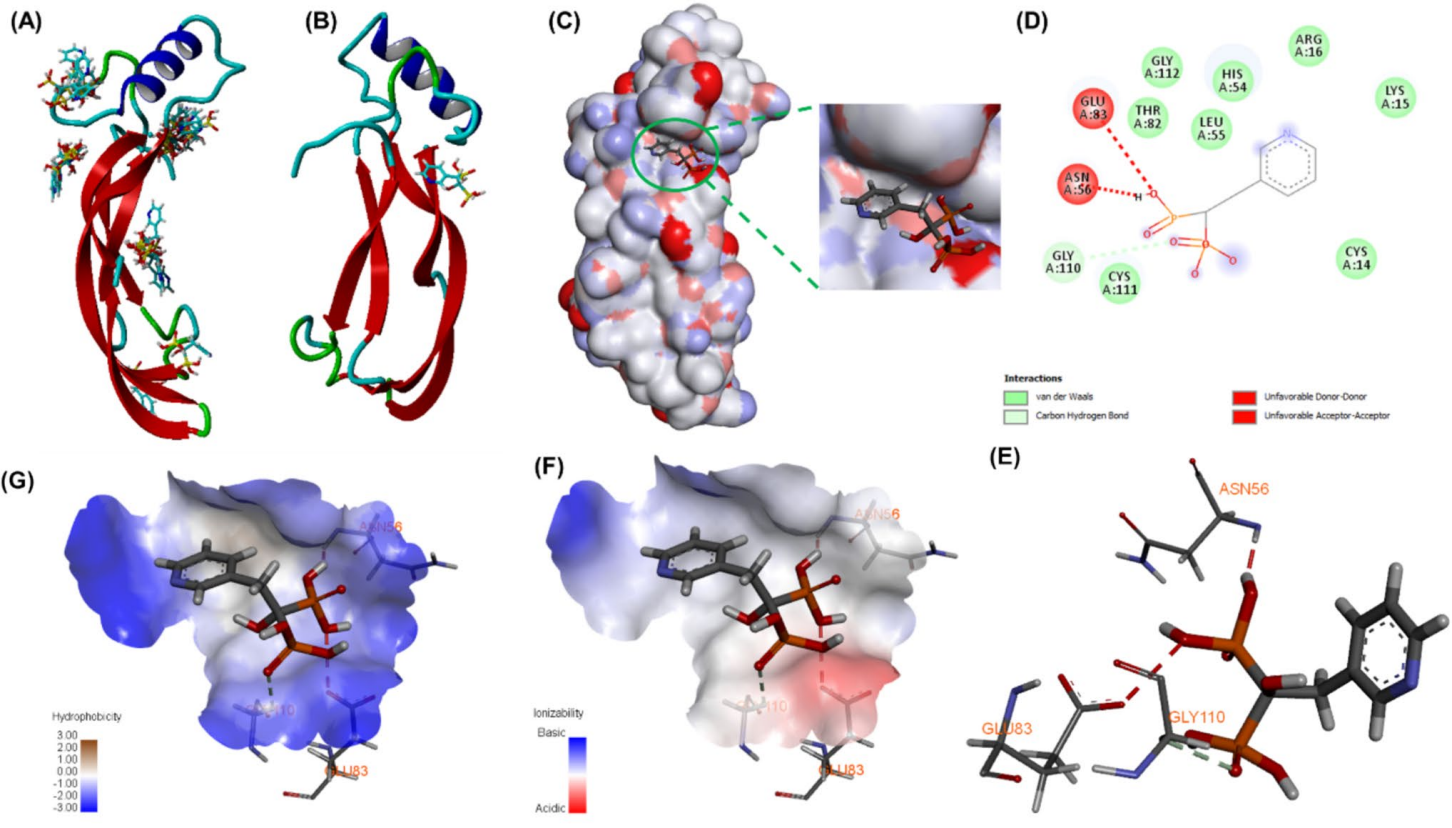
Molecular docking of BMP2–risedronate: **A** Cluster analysis with 25 runs. **B** stable binding confirmation of BMP2–risedronate. **C** Binding pockets of risedronate within the BMP2 rigid surface. **D**–**E** 2D and 3D binding conformation of the complex, and **F**–**G** Ionizability and hydrophobicity surface of the BMP2–risedronate binding pocket

Molecular docking and cluster analysis were performed to evaluate potential interactions between risedronate and BMP2. Cluster analysis with 25 random runs was conducted to determine the most probable interactions with the BMP2 backbone. As shown in Fig. 3A, B, the most conformations with the highest binding probability were selected for further analysis of binding interactions and binding energy. Similarly, the rigid BMP2 structure with the bound risedronate binding is shown in Fig. 3C, indicating that the binding pocket was not fully occupied by risedronate. Analysis of two-dimensional (2D) and three-dimensional (3D) amino acid interactions revealed that Asn56 and Glu83 formed unfavorable donor–acceptor bonds, whereas Gly110 engaged in a carbon–hydrogen bond interaction, with a binding energy of –5.1 kcal/mol (Fig. 3D, E). The re-docking study showed a binding energy of –4.2 kcal/mol. These results suggest that the interaction between risedronate and BMP2 is not highly stable. Additionally, examination of the hydrophobicity and ionization of the binding pocket indicated that the complex may dissociate under physiological conditions, particularly in slightly acidic or neutral hydrophilic environments, as bond contraction is more favorable in the less hydrophobic region and moderate acidic conditions (Fig. 3F, G). Collectively, these observations suggest that the binding between risedronate and BMP2 may be readily reversible under physiological conditions.

### Appraisal of BMP2—risedronate complex conformation stability using MD simulation

**Fig. 4:**
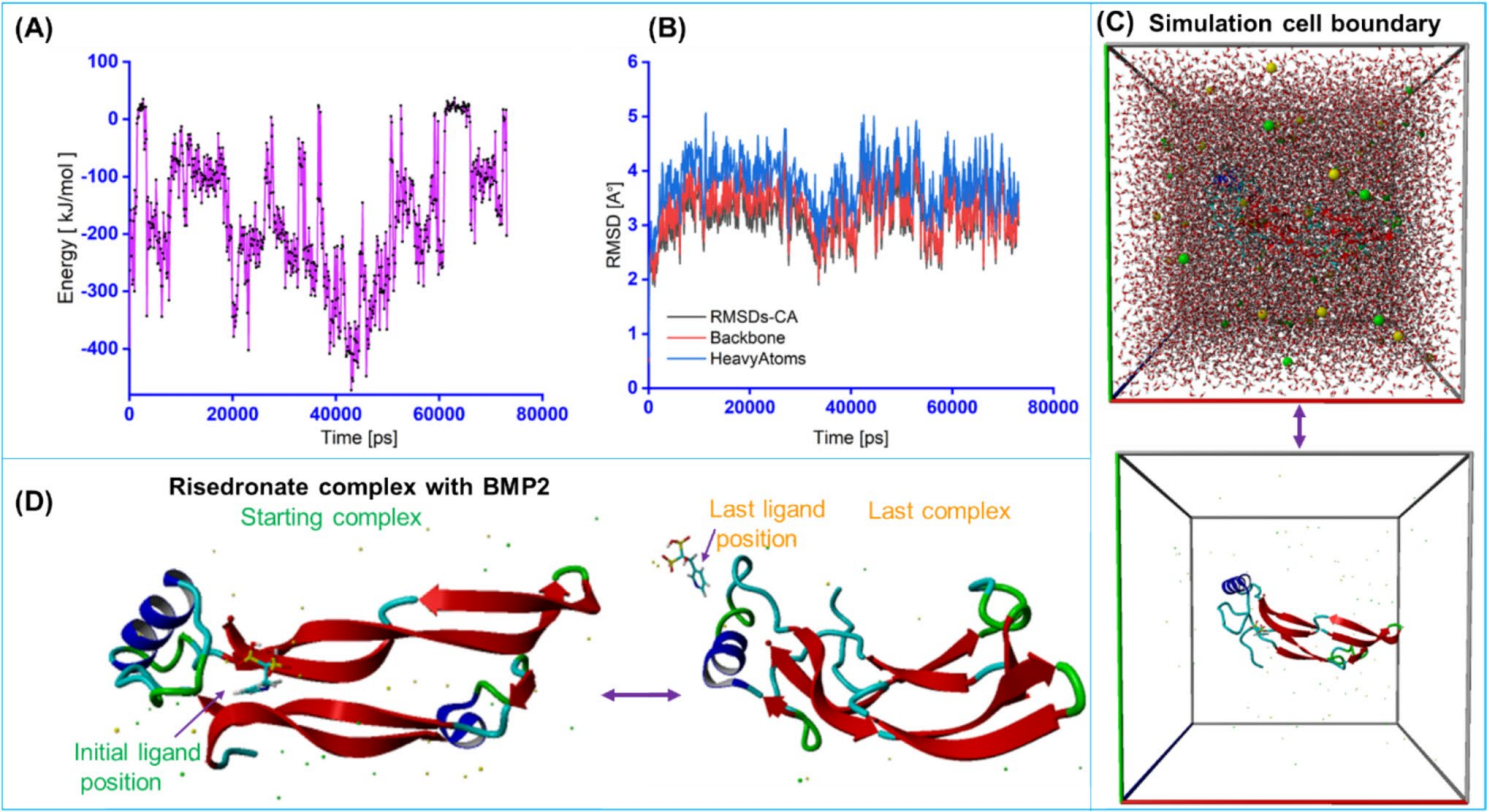
Stability assessment of the BMP2–risedronate ionic complex during MD simulation. **A**–**B** Comparative binding positions of the BMP2–risedronate ionic complex before and after extended runs of MD simulation. **C**–**D** RMSD and binding energy profiles of the BMP2–risedronate ionic complex

An in silico MD simulation approach was performed under physiological conditions to evaluate the conformation stability and motion of the BMP2–risedronate ionic complex. The analysis revealed an average binding energy of –172.04 kJ/mol, suggesting that the complex did not exhibit strong conformation binding between risedronate and BMP2 (Fig. 4A, B). Furthermore, according to the YASARA protocol, negative energy values in the graph represent unfavorable conformation instability, whereas positive energy values indicate favorable conformational stability. The BMP2—risedronate complex exhibited high fluctuation in binding energy during the MD run, ranging from 20 to –500 kJ/mol, confirming poor binding stability under physiological conditions over a 73.1 ns simulation.

As shown in Fig. 4C, the RMSD of the protein backbone and heavy atom fluctuated between 2 and 5 Å throughout the 73.1 ns MD run. Additionally, the initial and final ligand positions were notably dissimilar within the binding pocket (Fig. 4D). These findings indicate that, despite exhibiting stability, the BMP2–risedronate ionic complex may not remain stable under physiological conditions for extended periods. Consequently, partial dissociation of risedronate and BMP2 from the complex within the physiological environment is likely.

### In vitro BMP2 release profile from BMP2—risedronate ionic complex

**Fig. 5:**
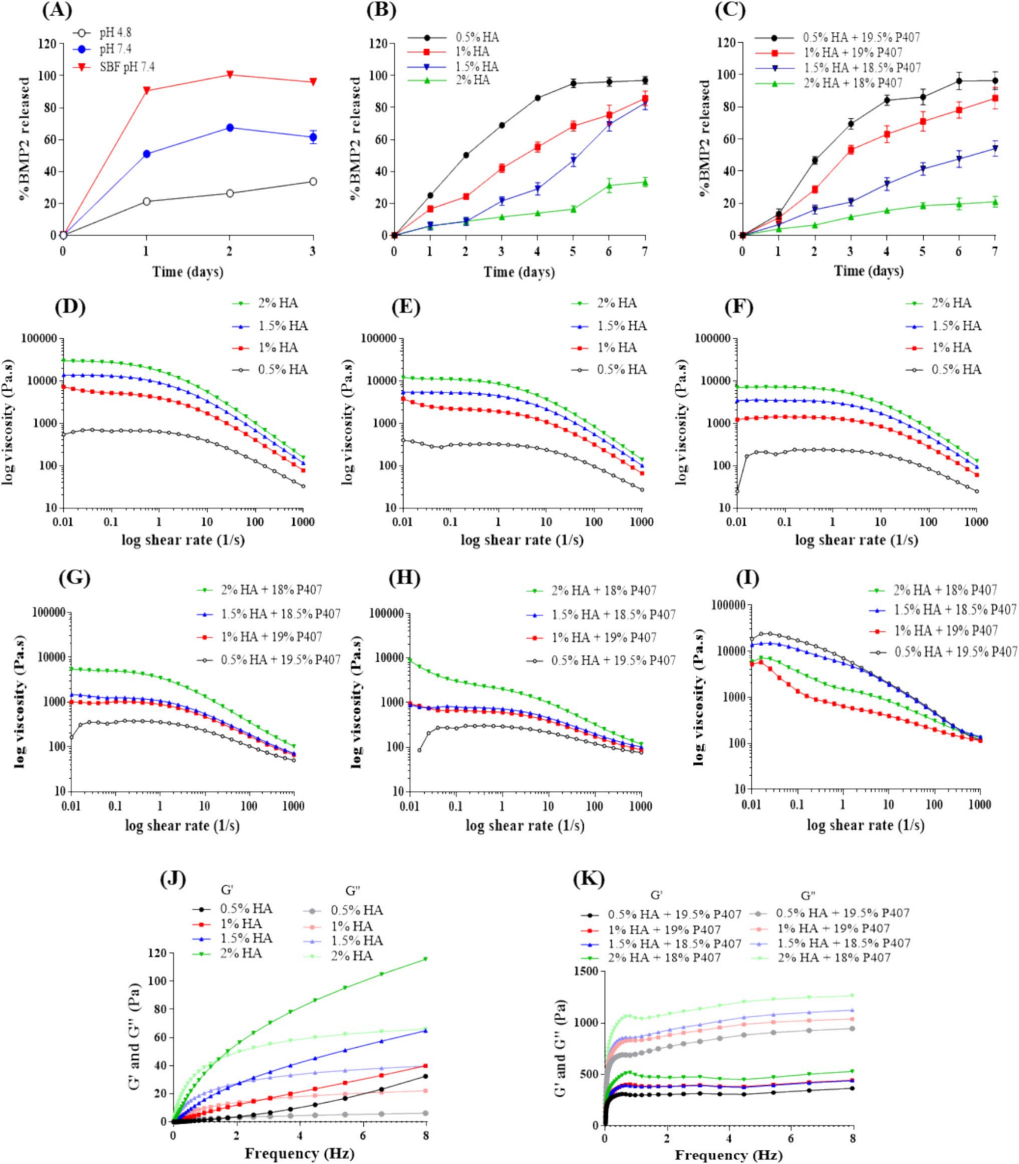
In vitro BMP2 release and rheological properties of hydrogels. **A** In vitro BMP2 release from the BMP2—risedronate ionic complex at pH 4.8, pH 7.4, and SBF pH 7.4. **B** In vitro BMP2 release from the BMP2—risedronate complex in hydrogels with varying HA concentrations. **C** BMP2 release from HA–P407 hybrid hydrogel. **D**–**F** Viscosity as a function of shear stress for HA solutions at 4 °C, RT, and 37 °C, respectively. **G**–**I** Viscosity as a function of shear stress for HA−P407 hybrid hydrogels at 4 °C, RT, and 37 °C, respectively. **J**–**K** Storage modulus (G') and loss modulus (G") as a function of frequency for HA and HA–P407 hybrid hydrogels, respectively

The amount of BMP2 released from the BMP2—risedronate ionic complex under various release media over 3 days is shown in Fig. 5A. In SBF (pH 7.4), BMP2 release was more rapid than at pH 4.8, with complete release occurring within 3 days. At pH 4.8, approximately 33% of BMP2 was released, whereas at pH 7.4, the release reached approximately 61% within the same period. Furthermore, the release profiles of BMP2 from BMP2—risedronate complex incorporated into hydrogels with varying concentrations of HA, with and without P407, over 7 days are shown in Fig. 5B, C. In the absence of P407 (Fig. 5B), BMP2 release decreased as HA concentration increased. The 0.5% HA released approximately 80% of BMP2 within 3 days and approximately 100% by day 7. The 1% HA exhibited a more controlled release, with approximately 50% BMP2 released by day 3 and 85–90% by day 7. The 1.5% and 2% HA demonstrated progressively slower release profiles, with the 1.5% hydrogel releasing approximately 30% BMP2 by day 3 and 70–75% by day 7, whereas the 2% hydrogel released less than 20% by day 3 and approximately 50% by day 7. In the presence of P407 (Fig. 5C), the release profiles followed a similar trend, with increasing HA concentrations leading to slower release rates. The 0.5% HA containing 19.5% P407 released BMP2 more rapidly, achieving approximately 100% release by day 7. The 1% HA with 19% P407 released approximately 60% BMP2 by day 3 and 90–95% by day 7. The 1.5% HA with 18.5% P407 exhibited a slower release rate, with approximately 40% released by day 3 and approximately 80% by day 7. The 2% HA with 18% P407 showed the slowest release, with less than 30% BMP2 released by day 3 and approximately 65% by day 7. Furthermore, the in vitro release data of the optimized BMP2 formulations (BMP2 ionic complex in 1.5% HA and BMP2 complex in 1% HA + 19% P407) were fitted to various kinetic models, including zero-order, first-order, Higuchi, Hixson-Crowell, and Korsmeyer-Peppas equations. The correlation coefficients (R^2^) and model parameters are summarized in Table 2**.** According to the analysis, the release profile of the formulation containing 1.5% HA did not fit any of the applied mathematical models, resulting in poor correlation coefficients across all tested kinetics. In contrast, the formulation containing 1% HA combined with 19% P407 exhibited a better model fit, with strong correlation coefficients for both first-order and Hixson–Crowell models, indicating that drug release was influenced by concentration-dependent kinetics and matrix erosion behavior.

**Table 2:** Kinetic modeling of BMP2 release from different hydrogel formulations Correlation coefficients (R^2^) for zero-order, first-order, Higuchi, Hixson–Crowell, and Korsmeyer-Peppas models

Formulations	Zero-order	First-order	Higuchi	Hixson-Crowell	Korsmeyer-Peppas
1.5% HA	0.9238	0.8255	0.7331	0.8629	0.8183
1% HA + 19% P407	0.9633	0.9870	0.9209	0.9855	0.9830

### Rheological characteristics of HA and HA–P407 hybrid hydrogels

The viscosity profiles of HA and HA–P407 hybrid hydrogels at 4 °C, RT, and 37 °C are shown in Fig. 5. All HA solutions exhibited shear-thinning behavior, with viscosity decreasing as shear rate increased. At 4 °C, HA solutions showed the highest viscosity, indicating greater resistance to flow at lower temperatures (Fig. 5D−F). At RT, viscosity decreased relative to 4 °C, while maintaining shear-thinning characteristics. At 37 °C, viscosity was lowest, reflecting minimal flow resistance under physiological conditions. In contrast, HA−P407 hybrid hydrogels exhibited the lowest viscosity at 4 °C, which increased with temperature, demonstrating the thermosensitive properties of P407 (Fig. 5G−I). At 37 °C, he formulation containing 0.5% HA and 19.5% P407 exhibited the highest viscosity, highlighting the contribution of P407 concentration to the enhanced flow resistance under physiological conditions. Similarly, the rheological properties of HA and HA–P407 hybrid hydrogels were evaluated by measuring the storage modulus (*G*′) and loss modulus (*G*′′) at various concentrations. For HA solutions (Fig. 5F), both *G*′ and *G*′′ increased with increasing HA concentration, indicating improved mechanical strength and viscoelastic behavior. Across all concentrations, *G*′ consistently exceeded *G*′′, reflecting the elastic, gel-like nature of the hydrogels. At lower concentrations, the moduli showed frequency dependence, suggesting a less stable network, whereas higher concentrations exhibited frequency-independent *G*′, indicative of greater stability. In contrast, the HA−P407 hybrid hydrogels (Fig. 5G) demonstrated significantly higher *G*′ and *G*′′ than HA-only formulations, reflecting enhanced mechanical properties and elasticity. The hybrid hydrogels exhibited enhanced stability at higher frequencies, which can be attributed to the synergistic effect of P407 in reinforcing the hydrogel network. Both *G*′ and *G*′′ increased with concentration, and the moduli reached a plateau at high frequencies, indicating the formation of a well-structured hybrid network.

### Injectability study of HA and hybrid hydrogels

The injectability of various hydrogel formulations was assessed at 4 °C and RT to determine their suitability for clinical application (Table Table 3 Injectability of various hydrogel formulations at 4 °C and RTSamplesInjectability (%)4 ℃RT0.5% HA1001001% HA1001001.5% HA1001002% HA1001000.5% HA + 19.5% P4071001001% HA + 19% P4071001001.5% HA + 18.5% P4071001002% HA + 18% P407100100^3^). All tested formulations, including HA (0.5%, 1%, 1.5%, and 2%) and hybrid hydrogels (0.5% HA + 19.5% poloxamer, 1% HA + 19% poloxamer, 1.5% HA + 18.5% poloxamer, and 2% HA + 18% poloxamer), exhibited 100% injectability at both 4 °C and RT, indicating excellent extrudability under these conditions. These findings demonstrate that all hydrogel formulations are highly injectable and suitable for in vivo administration under standard conditions.

**Table 3:** Injectability of various hydrogel formulations at 4 °C and RT

Samples	Injectability (%)	
	4 °C	RT
0.5% HA	100	100
1% HA	100	100
1.5% HA	100	100
2% HA	100	100
0.5% HA + 19.5% P407	100	100
1% HA + 19% P407	100	100
1.5% HA + 18.5% P407	100	100
2% HA + 18% P407	100	100

### Effect of BMP2—risedronate ionic complex on cell viability and in vitro osteogenic activity

**Fig. 6:**
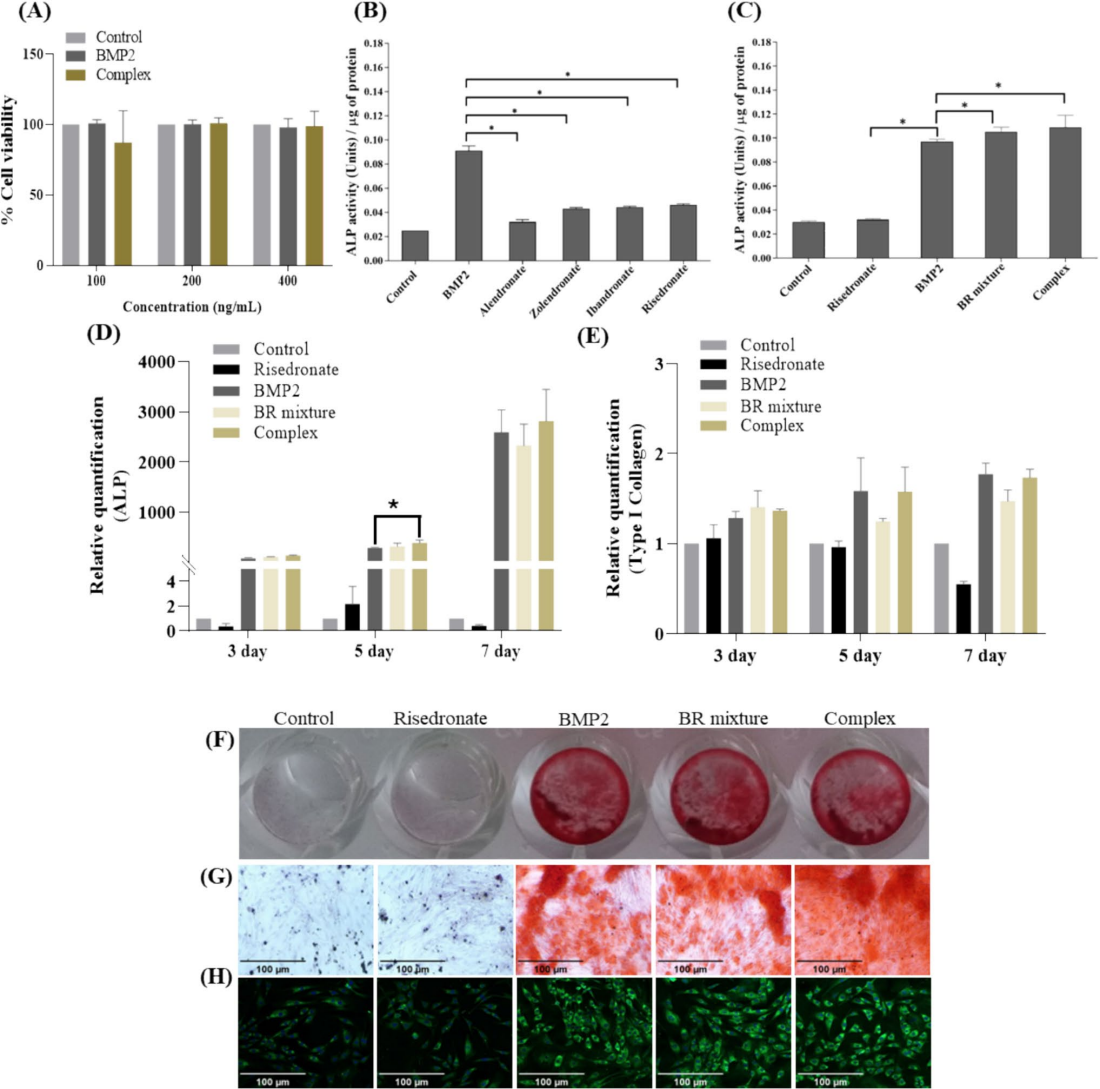
In vitro cellular studies. **A** Cell viability of C2C12 cells after treatment with different BMP2 formulations. **B** In vitro ALP activity after treatment with BMP2 and bisphosphonates. **C** ALP activity after treatment with BMP2, risedronate, BMP2—risedronate mixture, and BMP2–risedronate ionic complex. Expression of mRNA associated with osteoblastic differentiation marker following treatment with various formulations: ALP (**D**) and type I collagen (*p < 0.05). **E** Alizarin Red S staining and immunofluorescence assay for osteogenic differentiation. **F** Microplate photographs. **G** Microscopic images of Alizarin Red S staining. **H** Immunofluorescence imaging of type I collagen expression. In all panels, the images from left to right correspond to control, risedronate, BMP2, BMP2—risedronate mixture, and BMP2—risedronate complex, respectively

Before evaluating the osteoblastic differentiation capability of the BMP2–risedronate ionic complex, cell viability was assessed following treatment with BMP2 and BPs alone to examine cytotoxicity and determine optimal concentration for both components. No cytotoxicity was observed for BMP2 or the BMP2–risedronate ionic complex at concentrations ranging from 100 to 400 ng/mL (Fig. 6A). Among the four BPs tested, including alendronate, ibandronate, zoledronate, and risedronate, cell viability varied with concentration. At concentrations above 0.5 μM, alendronate and zoledronate reduced cell viability to below 80%, whereas ibandronate and risedronate showed no cytotoxicity within the range of 0.1–1 μM (Fig. S3).

ALP activity, a key osteoblast-specific marker, was evaluated to assess the osteoblastic differentiation of C2C12 myoblasts. The concentrations of both components were determined based on the cell viability assay results to facilitate the evaluation of ALP activity in C2C12 cells. Cells were incubated with BMP2 at an equivalent concentration of 300 ng/mL, and BPs were administered at 0.1 μM (alendronate and zoledronate), 1 μM (ibandronate), and 0.3 μM (risedronate). Following the cell viability assay, ALP activity was measured to determine the stimulatory effect of BMP2 in combination with the four BPs on C2C12 cells. Although BPs alone did not markedly increase ALP activity compared with BMP2 alone, risedronate exhibited a higher ALP activity than the other BPs (Fig. 6B). Based on this observation, risedronate was selected as the preferred counterpart for the preparation of the BMP2–risedronate ionic complex. The in vitro bioactivity of the BMP2—risedronate ionic complex was further examined by measuring ALP activity in C2C12 cells after 7 days of differentiation. The BMP2 released from the BMP2—risedronate ionic complex retained its osteoinductive potential, successfully inducing the differentiation of C2C12 myoblasts into osteoblasts (Fig. 6C). Notably, the BMP2—risedronate ionic complex significantly increased ALP activity compared with BMP2 alone at an equivalent concentration.

The osteogenic activity of BMP2 released from the BMP2—risedronate ionic complex in C2C12 cells was confirmed by measuring mRNA expression levels of a molecular marker associated with the early stages of osteoblastic differentiation. As shown in Fig. 6D, E, treatment with risedronate alone did not induce ALP mRNA expression and reduced type I collagen mRNA expression compared with the negative control in a time-dependent manner. However, the BMP2—risedronate ionic complex exhibited higher ALP mRNA expression than BMP2 alone.

Alizarin Red S staining was performed to evaluate the mineralization capacity of BMP2 formulations during osteoblastic differentiation of C2C12 cells. Microscopic images showed no mineralization in the control and risedronate-treated group. In contrast, treatment with the BMP2—risedronate ionic complex resulted in a marked increase in mineralization compared with the BMP2-treated group (Fig. 6F, G).

In addition, immunocytochemistry was conducted to examine the effect of the BMP2—risedronate ionic complex and its mixtures on type I collagen expression during the osteoblastic differentiation of C2C12 cells. As shown in Fig. 6H, compared with the control group, type I collagen expression was higher in cells treated with the BMP2—risedronate ionic complex than in those treated with BMP2 alone, the BMP2—risedronate mixture, or dissociated forms. These findings are consistent with type I collagen mRNA expression results shown in Fig. 6E.

### In vivo osteogenic efficacy of BMP2—risedronate ionic complex

**Fig. 7:**
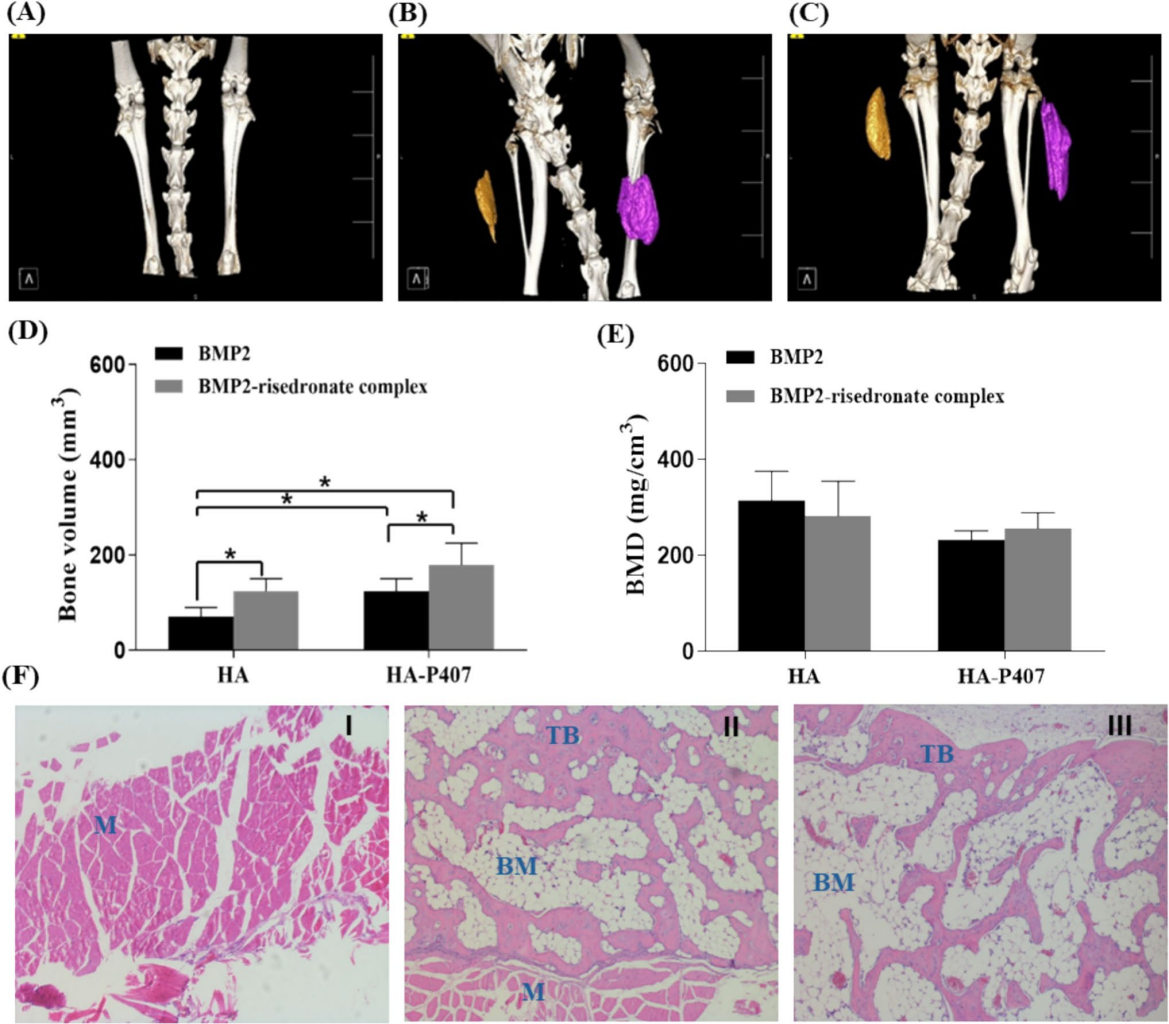
Representative 3D images of in vivo ectopic bone formation in the control group (**A**), hydrogel-loaded BMP2 treatment group (**B**), and hydrogel-loaded BMP2—risedronate complex treatment group (**C**). Different colors indicate the newly formed bones: yellow for HA and purple for HA–P407 hybrid hydrogel. Bone volume (mm^3^) was quantified using micro-CT analysis under HA and HA–P407 hybrid hydrogel (**D**). Bone mineral density (mg/cm^3^) was determined using micro–CT analysis under HA and HA−P407 hybrid hydrogel (**E**). Representative histological images of ectopic bone formed by intramuscular implantation (H&E stain, 100×) (**F**) are shown for the control group (I), BMP2 group (II), and complex group (III). Photomicrographs demonstrate mature TB containing active osteocytes and hematopoietic marrow (BM). The newly generated bone was surrounded by skeletal muscle (M)

We investigated an enhanced osteogenic efficacy achieved by the sustained release of BMP2 and the stimulatory effect of risedronate using an in vivo ectopic bone formation model. After 4 weeks of implantation with HA-based hydrogel and HA−P407 hybrid hydrogel containing BMP2–risedronate ionic complex, ectopic bone nodules were observed, and their total volume was quantified using μCT software. The μCT reconstruction images revealed ectopic bone formation in both the BMP2 group and the BMP2–risedronate ionic complex group (Fig. 7B, C) compared with the negative control group (Fig. 7A). Furthermore, transaxial and 3D CT images showed that the newly formed bone within the HA−P407 hybrid hydrogel (right hind limb) was larger than that in the HA hydrogel (left hind limb).

Following imaging, the net volume of the newly formed bones (BV, mm^3^) was measured by isolating the trabecular bone (TB) structure from the original bone marrow portion. In the BMP2–risedronate ionic complex groups, BV was higher than in the BMP2 group. Specifically, the BV in the BMP2–risedronate ionic complex group with HA was approximately 2.02-fold higher than that in the BMP2 group (*p* < 0.05). Moreover, the HA–P407 hybrid hydrogel system further enhanced bone formation, with BV in the BMP2–risedronate ionic complex group showing a significant increase compared with the BMP2 group. Notably, BV was highest in the HA–P407 hybrid hydrogel system, demonstrating its superior capacity to support bone formation and enhance the osteogenic efficacy of the BMP2–risedronate ionic complex (Fig. 7D).

The BMD of the newly generated bone was determined using relative quantitative measurement of radiodensity (Hounsfield units, HU) from calibrated CT data. BMD was comparable between the HA and HA–P407 hybrid hydrogel groups, indicating that although the hybrid system promoted greater bone volume, the density of the newly formed bone remained consistent across both systems (Fig. 7E). To confirm ectopic bone formation, histological evaluation was performed on bone tissue sections stained with H&E. Control groups treated with BMP2-free hydrogel exhibited no bone formation (Fig. 7F–I). Both hydrogels were completely resorbed at the injection site. Except for the control groups, H&E staining revealed newly formed bone in all specimens. The injection sites were replaced with new TB containing active osteocytes and hematopoietic marrow, surrounded by host muscle tissue (Fig. 7F-II,-III). These findings were consistent with the transaxial μCT images.

## Discussion

This study is the first to report an ionic complex system using risedronate as a potential counterpart for the sustained release of BMP2, aimed at enhancing osteogenic efficacy in an ectopic bone formation model. Multiple analytical techniques were employed to characterize the BMP2–risedronate ionic complex after its successful formation. The concept of the BMP2–risedronate ionic complex system builds on findings from our previous research (Lee et al. 2010). The BMP2–risedronate ionic complex exhibited a mean particle size of approximately 326 ± 149 nm, with a PDI of 0.2–0.3, indicating a relatively uniform and monodisperse population. This nanoscale size range is particularly advantageous for biomedical applications, especially for local delivery and tissue penetration. In addition, the complex demonstrated a zeta potential of approximately − 38 mV, suggesting high colloidal stability due to sufficient electrostatic repulsion. The strong negative surface charge likely arises from the ionized phosphonate groups in risedronate, which remain deprotonated at physiological pH. This surface charge stabilizes the complex in aqueous conditions, minimizes nonspecific protein adsorption, and may modulate cellular interactions, particularly favoring accumulation in mineralized tissues. FT–IR spectra and hyperspectral images confirmed that the negatively charged risedronate participated in electrostatic interactions with the positively charged BMP2 to form a complex. Computational simulation conducted in solution under physiological conditions further supported the formation and stability of the complex. Molecular docking suggested that the interaction between risedronate and BMP2 is sufficiently strong to maintain complex stability, while allowing dissociation under hydrophilic conditions at moderate acidic or neutral pH, where bond contraction is energetically favorable (Fig. 3G and F). Furthermore, MD simulation revealed that the BMP2–risedronate ionic complex can dissociate under physiological conditions over an extended period.

A critical observation from the ALP activity assay was the enhanced differentiation of C2C12 cells in the presence of the BMP2–risedronate ionic complex and the BMP2—risedronate mixture compared with BMP2 alone. The ability of the complex to significantly upregulate ALP activity demonstrates its efficacy in inducing osteoblast differentiation. The retained bioactivity of BMP2 post-release underscores the structural integrity of the BMP2–risedronate ionic complex and its dissociation under physiological conditions. The mineralization potential, assessed via alizarin red staining, revealed clear evidence of calcium deposition in cells treated with the complex. Immunocytochemistry further corroborated these findings by demonstrating increased expression of type I collagen. Collectively, these results highlight the osteogenic efficacy of the BMP2–risedronate ionic complex, confirming its ability to preserve BMP2 bioactivity.

Collagen is widely used as a delivery system for BMP2 due to its abundant availability, biodegradability, and role as a major component of the bone matrix (Mumcuoglu et al. 2018; Jeon et al. 2022). However, an initial burst release of BMP2 from collagen sponges has been reported due to their low retention capacity; approximately 50–80% of the loaded BMP2 is released within 24 h, with only 5% of BMP2 remaining 2 weeks post-implantation (Bhakta et al. 2012). To address this limitation and enhance osteogenic efficacy, researchers have focused on developing a novel carrier system that can prolong BMP2 release. Several studies have demonstrated that the release profile of BMP2 is closely related to the osteogenic efficacy. For instance, Bhakta et al. investigated the effect of carrier release kinetics on fracture healing efficacy (Bhakta et al. 2013). Their findings indicated that BMP2 released with a low initial burst followed by sustained release promoted significantly greater bone formation than BMP2 released in a sustained manner. These observations are consistent with previous reports (Olthof et al. 2019; Howard et al. 2022), suggesting that the extremes of release, such as bolus injections or prolonged low-level release, are suboptimal for bone formation. It is noteworthy that an initial burst release of BMP2 is necessary to recruit osteoprogenitor cells to the implantation site, whereas sustained release supports the differentiation of these cells into osteoblasts. Building on these insights, recent advances have highlighted ionic complexation as an effective strategy to modulate BMP2 release profiles. Ionic complexes, formed via electrostatic interactions between oppositely charged molecules, can create a low-solubility, high-affinity state that reduces the initial burst release while enabling sustained protein availability. Several studies have demonstrated that ion pairing alone, without the use of hydrogel matrices, can effectively prolong the release of BMP2. For example, heparin–polycation coacervate microparticles significantly reduce the initial burst and sustained BMP-2 over extended periods due to strong electrostatic interactions, which maintain the protein in a low-solubility, high-affinity state (Li et al. 2013). Similarly, PLGA nanoparticles with acidic end groups achieved slower BMP2 desorption through ion pairing, effectively preventing burst release even in the absence of a structural matrix (Del Castillo-Santaella et al. 2019).

This study aimed to address these challenges by utilizing a BMP2–risedronate ionic complex in combination with HA- and P407-based hybrid hydrogel systems, which demonstrated the capacity for controlled and sustained BMP2 delivery. Release studies of BMP2 from the BMP2–risedronate ionic complex under three different buffer conditions revealed a relatively faster release at physiological pH (PBS pH 7.4 and SBF pH 7.4) compared with acidic pH. Notably, at SBF 7.4, release was nearly complete within a single day. To mitigate this rapid release and enhance BMP2 retention at the injection site, HA and HA–P407-based matrices were incorporated to modulate release kinetics and maintain local availability. HA, a high-molecular-weight non-sulfated glycosaminoglycan (GAG), is a natural component of synovial fluid and cartilage, exhibiting excellent lubricating, shock-absorbing, and hydration properties. Clinically, HA has been used for soft tissue replacement and augmentation, as well as in surgical procedures and diagnostics (Lorenzi et al. 2024; Hur et al. 2025). HA has also been shown to enhance BMP2-mediated osteogenic and angiogenic activity of BMP2 in SD rats (Huang et al. 2017). P407 is a thermo-responsive polymer frequently employed in local injectable formulations due to its ability to reduce protein degradation and sustain therapeutic drug release (Ruiz et al. 2022; Marques et al. 2023). P407 possesses excellent water solubility and low toxicity, making it highly biocompatible (Yang et al. 2021). Although P407 does not inherently induce osteoinduction, its combination with BMP2 as a gel matrix has been shown to stimulate bone formation in defect areas (Tateiwa et al. 2021). To optimize the delivery system, various concentrations of HA and combinations of HA with different concentrations of P407 were tested. Based on rheological properties and release studies, 1.5% HA and a hybrid formulation of 1% HA with 19% P407 were identified as suitable candidates. Selection criteria focused on achieving structural stability and ensuring controlled BMP2 release to maintain its therapeutic efficacy. Mathematical modeling of in vitro release profiles provided critical insight into the mechanisms governing the release of BMP2. The 1.5% HA formulation failed to fit any tested kinetic models (zero-order, first-order, Higuchi, and Hixson–Crowell), as indicated by low correlation coefficients across the board, suggesting an irregular or non-uniform release pattern likely due to inadequate structural or network properties of the hydrogel. Higher HA concentrations may increase viscosity and cross-linking density, hindering consistent drug diffusion and contributing to erratic release. Additionally, HA alone lacks thermoresponsive behavior, limiting its capacity to modulate drug release in a controlled manner. In contrast, the formulation containing 1% HA and 19% P407 demonstrated significantly improved fit to both the first-order and Hixson–Crowell models, with higher correlation coefficients (R^2^ > 0.97). The first-order fit indicates concentration-dependent BMP2 release, characteristic of systems where diffusion predominates. The Hixson–Crowell model, which accounts for changes in surface area and geometry during dissolution, suggests that matrix erosion or shrinkage also contributes to release. The synergistic effect between HA and P407 enhances the hydrogel’s structural integrity and responsiveness, forming a semi-solid matrix that enables sustained release via a combination of diffusional transport and matrix relaxation or erosion. The in vivo results revealed a marked difference in bone formation activity between the two delivery systems. The incorporation of P407 into the HA matrix increased osteogenic efficacy compared with HA alone under identical conditions, suggesting that the hybrid hydrogel system enhances retention of the BMP2–risedronate ionic complex at the injection site and synergistically promotes osteogenesis. Additionally, the presence of risedronate contributed to enhanced bone formation, consistent with previous findings that bisphosphonates promote osteogenesis partly through osteoclast inhibition (Ke et al. 2021; Xu et al. 2021a). Prior studies involving local administration of BMP2 in combination with BPs such as zoledronate, minodronate, and pamidronate using matrices including collagen–hydroxyapatite scaffolds, tricalcium phosphate, and poly-D, L-lactic-acid pellets have similarly demonstrated bone formation (Murphy et al. 2014; Oryan et al. 2023; Hong et al. 2025). Furthermore, the presence of BPs has been reported to increase bone formation, which may be partly due to osteoclast-inhibition activity (Wang et al. 2024a, 2024b; Hadad et al. 2025). In this study, the stimulatory effect of risedronate on BMP2-mediated osteogenesis was demonstrated using an ectopic bone formation animal model. In both the HA and HA−P407 hybrid hydrogel groups, the BV value of the BMP2—risedronate ionic complex increased significantly compared with BMP2 alone. While BMP2, directly and indirectly, induces both osteoclast and osteoblast differentiation, many osteoclasts can be observed in newly induced bone tissue when stained with tartrate-resistant acid phosphatase after inoculation of BMP2 into muscle (Bordukalo-Nikšić et al. 2022b; Wang et al. 2023). However, in the presence of risedronate, osteoclasts differentiated by BMP2 inoculation may be inactivated, resulting in enhanced osteogenic efficacy.

Although appropriate BP dosing is critical, reports on local implantation of risedronate are limited. To our knowledge, this study is the first to demonstrate that local implantation of risedronate, an ionic complex system with BMP2, exerts a synergistic effect on bone formation. Additionally, the effect of different hydrogel matrices on osteogenic potency was investigated, revealing that the combination of HA with P407 further enhanced BMP2 activity. Optimization of risedronate dosage and selection of a suitable matrix capable of retaining the drug allows for the development of advanced BMP2 delivery systems for effective bone augmentation.

The findings of this study indicate that the BMP2–risedronate ionic complex is a promising candidate for local BMP2 delivery with improved osteogenic efficacy. Ion pairing of BMP2 with risedronate, a third-generation bisphosphonate, produced uniformly distributed, nanosized particles while preserving BMP2 secondary structure integrity under the experimental conditions. Furthermore, in vitro release studies of the BMP2–risedronate ionic complex demonstrated a controlled release profile of BMP2 over 7 days across different release media, which corresponded with enhanced osteoblastic differentiation of C2C12 myoblasts in vitro and improved in vivo osteogenic efficacy in SD rats compared with BMP2 alone. Collectively, these results support the hypothesis that the BMP2–risedronate ionic complex enables sustained release of BMP2 and effectively promotes osteogenesis. This system addresses limitations associated with burst release from conventional collagen-based delivery systems and offers a promising strategy for efficient growth factor delivery in clinical applications.

To facilitate translation, scalable manufacturing methods must be optimized to preserve complex stability and biological activity during production and storage. Regulatory approval will necessitate a thorough evaluation of dosing, batch consistency, and product safety, taking into account the known risks of ectopic bone formation and potential adverse effects associated with prolonged exposure to BMP2. Moreover, comprehensive in vivo studies will be essential to assess efficacy, biodistribution, and safety profiles, providing a foundation for eventual clinical application.

## Supplementary Information

Below is the link to the electronic supplementary material.

Supplementary file1 (DOCX 555 KB)

## Data Availability

The datasets generated and analyzed during the current study are available from the corresponding author upon reasonable request.
