# Supplementary material for: Engineering a BMP2–risedronate complex with sustained release for osteoporosis therapy
Source: Arch Pharm Res. 2025 Sep 24;48(9-10):1001–21. doi: 10.1007/s12272-025-01568-8 (PMC12589347; doi:10.1007/s12272-025-01568-8)
Supplement: Supplementary file 1 — Supplementary file1 (DOCX 555 KB) [file 12272_2025_1568_MOESM1_ESM.docx]

Supporting Information


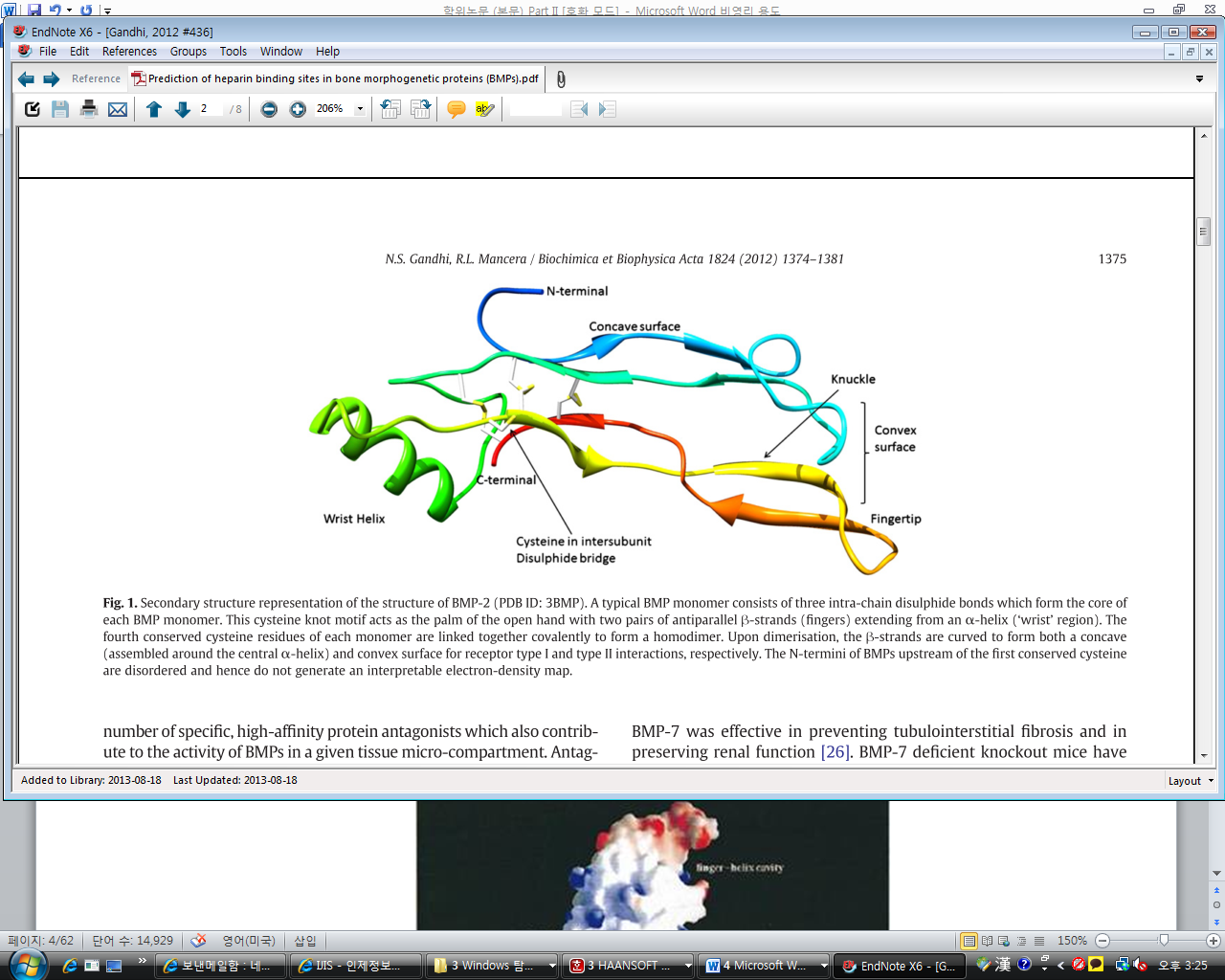


(A)





(B)

**Figure S1.** Structure of BMP2. (A) Secondary structure representation of the BMP2 and (B) solvent-accessible surface representation of BMP-2. Red color indicates negative surface charge, and blue color indicates positive surface charge. White color indicates hydrophobic regions.

**
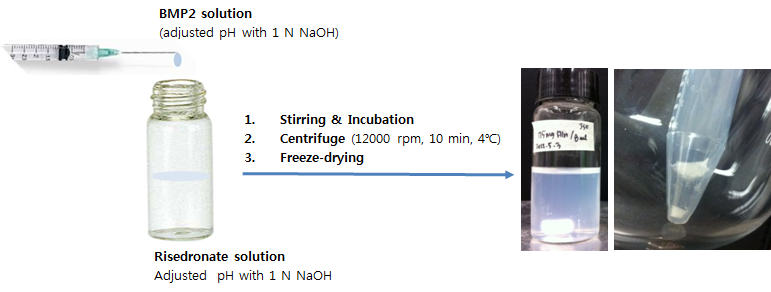
Figure S2**. Scheme of BMP2-risedronate ionic complex preparation.

**Figure S3**. Cell viability of C2C12 cell after treatment of four types of bisphosphonates.
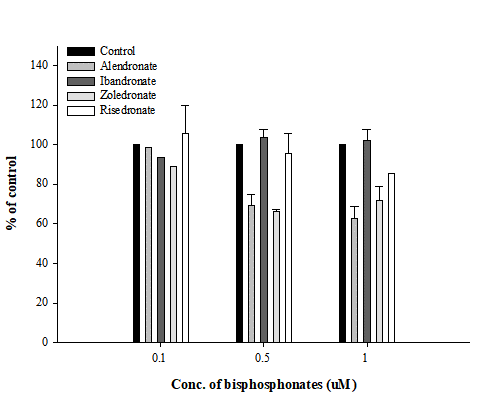


**Table S1.** Primer sequences used for relative quantification of osteoblastic differentiation gene in this study

| **Target Gene** | **Primer Sequence (5′ to 3′)** | |
| --- | --- | --- |
| ALP | Forward | ATGGGCGTCTCCACAGTAAC |
|  | Reverse | TCACCCGAGTGGTAGTCACA |
| Type I collagen | Forward | ACGTCCTGGTGAAGTTGGTC |
|  | Reverse | CAGGGAAGCCTCTTTCTCCT |
| GAPDH | Forward | AACTTTGGCATTGTGGAAGG |
|  | Reverse | ACACATTGGGGGTAGGAACA |
